# *Stenotrophomonas maltophilia* responds to exogenous AHL signals through the LuxR solo SmoR (Smlt1839)

**DOI:** 10.3389/fcimb.2015.00041

**Published:** 2015-05-15

**Authors:** Paula Martínez, Pol Huedo, Sònia Martinez-Servat, Raquel Planell, Mario Ferrer-Navarro, Xavier Daura, Daniel Yero, Isidre Gibert

**Affiliations:** ^1^Grup de Genètica Molecular i Patogènesi Bacteriana, Institut de Biotecnologia i de Biomedicina, Universitat Autònoma de BarcelonaBarcelona, Spain; ^2^Departament de Genètica i de Microbiologia, Universitat Autònoma de BarcelonaBarcelona, Spain; ^3^Catalan Institution for Research and Advanced StudiesBarcelona, Spain

**Keywords:** LuxR Orphan, AHL, Acyl-Homoserine lactone, lactonase, quorum sensing, swarming

## Abstract

Quorum Sensing (QS) mediated by Acyl Homoserine Lactone (AHL) molecules are probably the most widespread and studied among Gram-negative bacteria. Canonical AHL systems are composed by a synthase (LuxI family) and a regulator element (LuxR family), whose genes are usually adjacent in the genome. However, incomplete AHL-QS machinery lacking the synthase LuxI is frequently observed in Proteobacteria, and the regulator element is then referred as LuxR solo. It has been shown that certain LuxR solos participate in interspecific communication by detecting signals produced by different organisms. In the case of *Stenotrophomonas maltophilia*, a preliminary genome sequence analysis revealed numerous putative *luxR* genes, none of them associated to a *luxI* gene. From these, the hypothetical LuxR solo Smlt1839, here designated SmoR, presents a conserved AHL binding domain and a helix-turn-helix DNA binding motif. Its genomic organization—adjacent to *hchA* gene—indicate that SmoR belongs to the new family “LuxR regulator chaperone HchA-associated.” AHL-binding assays revealed that SmoR binds to AHLs *in-vitro*, at least to oxo-C8-homoserine lactone, and it regulates operon transcription, likely by recognizing a conserved palindromic regulatory box in the *hchA* upstream region. Supplementation with concentrated supernatants from *Pseudomonas aeruginosa*, which contain significant amounts of AHLs, promoted swarming motility in *S. maltophilia*. Contrarily, no swarming stimulation was observed when the *P. aeruginosa* supernatant was treated with the lactonase AiiA from *Bacillus subtilis*, confirming that AHL contributes to enhance the swarming ability of *S. maltophilia*. Finally, mutation of *smoR* resulted in a swarming alteration and an apparent insensitivity to the exogenous AHLs provided by *P. aeruginosa*. In conclusion, our results demonstrate that *S. maltophilia* senses AHLs produced by neighboring bacteria through the LuxR solo SmoR, regulating population behaviors such as swarming motility.

## Introduction

Bacterial cells can communicate with each other to facilitate their rapid adaptation to fluctuations in the environment. This cell-cell communication mechanism, known as quorum sensing (QS), relies primarily on the production, detection, and response to diffusible signal molecules (also called autoinducers) in a cell-density dependent manner (Fuqua et al., 1994; Whitehead et al., 2001; Fuqua and Greenberg, 2002; Federle and Bassler, 2003). Through this QS communication, numerous bacterial species regulate a variety of functions such as biofilm formation, motility, antibiotic resistance, toxin production, exopolysaccharide synthesis, and extracellular enzyme production among others (Miller and Bassler, 2001). In Gram-negative bacteria, *N*-acyl homoserine lactones (AHLs) are to date the most extensively and best characterized QS signaling molecules. AHL-QS regulation consists of a LuxI-type synthase, which produces signal molecules, and a LuxR-type receptor that binds AHLs and regulates expression of certain genes when signal concentration reaches a critical threshold. LuxR regulators are about 250 residues in length and present two typical domains, the N-terminal autoinducer AHL binding domain (Shadel et al., 1990; Slock et al., 1990) and the C-terminal helix-turn-helix (HTH) DNA-binding domain (Choi and Greenberg, 1991; Fuqua and Winans, 1994). In the presence of AHLs, the N-terminal binding domain interacts with the signal molecule, habilitating the DNA-binding domain to induce transcription of certain genes by binding to their promoters in a region named *luxR* box (Devine et al., 1989; Stevens and Greenberg, 1997). The DNA-binding domain includes three highly conserved aminoacids, while the AHL-binding domain presents six hydrophobic or aromatic residues displaying remarkable variability (18-25%) (Zhang et al., 2002).

The increasing availability of bacterial genome sequences has led to the identification of several LuxR and LuxI homologs. Typically, both *luxI*-type and *luxR*-type genes are located adjacent in the bacterial genome (the cognate *luxR/I* pair). However, *luxR*-type genes without a cognate *luxI*-type in their vicinity are frequently found, and these regulatory elements are then called “orphan” (Fuqua, 2006) or “solo” LuxR (Subramoni and Venturi, 2009a). Recent studies have revealed that “*luxR* solo” genes are widely distributed among bacterial genomes (Hudaiberdiev et al., 2015). LuxR solos present the same modular organization as canonical LuxR, displaying the N-terminal and C-terminal domains. The nature of the signal molecules that bind to the different LuxR solos is quite heterogeneous. It has been shown that the LuxR solo QscR from *Pseudomonas aeruginosa* bind to self-produced AHL signals (Chugani et al., 2001; Lequette et al., 2006), while SdiA of *Salmonella enterica* and *Escherichia coli* respond to exogenous AHL signals (Ahmer et al., 1998; Michael et al., 2001; Ahmer, 2004; Yao et al., 2006). Interestingly, the LuxR solo OryR from *Xanthomonas oryzae* pv. *oryzae* interacts with plant signals, in particular those produced by rice (Ferluga et al., 2007; Ferluga and Venturi, 2009; González et al., 2013). More recently, it has been reported that the human and insect pathogen *Photorhabdus asymbiotica* contains a LuxR solo PauR that is the regulator element of a new QS-system, which is mediated by dialkylresorcinols (DARs) and cyclohexanediones (CHDs) signals (Brameyer et al., 2015). Altogether, this shows that LuxR solos can participate in a wide variety of signaling networks.

*Stenotrophomonas maltophilia* is an ubiquitous gram-negative bacterium considered an emerging nosocomial pathogen (Brooke, 2012). Moreover, it is frequently found in lungs of cystic-fibrosis (CF) patients (Demko et al., 1998), usually co-isolated with *P. aeruginosa* (Moskowitz et al., 2005). The QS described in *S. maltophilia* is based on the signaling molecule DSF (11-cis-2-decenoic acid), by which it regulates virulence-related processes (Fouhy et al., 2007; Huedo et al., 2014). To date, no *S. maltophilia* strain has been reported to produce AHL and the K279a reference genome contains no *luxI* homolog (Crossman et al., 2008), at least of the usual *luxI* types (Waters and Bassler, 2005). However, sequence analysis reveals that this genome encodes a total of 15 putative LuxR-like proteins, based mainly on homologies of the DNA-binding response domain. From these, only the LuxR solo Smlt1839 showed an N-AHL autoinducer-binding domain. The objective of our study has been to experimentally investigate the role of Smlt1839 in AHL binding and swarming regulation in *S. maltophilia*, in the presence of exogenous and heterologous AHLs.

## Materials and methods

### Bacterial strains and growth conditions

Bacterial strains used in this study are listed in Table 1. *E. coli* strains DH5a and BL21 (DE3) were used for general cloning purposes and overexpression of *smoR*, respectively. *S. maltophilia* E77 (Ferrer-Navarro et al., 2013) was used as a model strain to investigate the role of SmoR in detection and response of exogenous AHL signals. *P. aeruginosa* MPAO1 strain was used as an AHL-producer bacterium to evaluate the effect of exogenous signal molecules on swarming motility of *S. maltophilia*. *Agrobacterium tumefaciens* KYC55 (Zhu et al., 2003) was used as a reporter strain to detect AHL production.

**Table 1:** **Primers used in this study**.

**Primer**	**Sequence (5′-3′)**	**Restriction site**
P1MutSmoR	AAGCTTTGCCCGGTTCGGTATCGG	*Hind*III
P2MutSmoR	GGATCCTCGCGGCGAGGCACTTCC	*BamH*I
P3MutSmoR	GGATCCCCGGTTCAGCGCCCGGCC	*BamH*I
P4MutSmoR	GAATTCCCCAGCCGCCAGCCCAGC	*EcoR*I
PErm5′	GGATCCGAAACGTAAAAGAAGTTATG	*BamH*I
PErm3′	GGATCCTACAAATTCCCCGTAGGC	*BamH*I
PErm5′rev	GATACTGCACTATCAACACAC	–
PErm3′rev	CTTCCAAGGAGCTAAAGAGGT	–
P1DemSmoR	GTACGTCGGGCGTATCG	–
P2DemSmoR	GCCCTTCTATGCTGG	–
P1ProSmoR	TCTAGACGCACACGCATGGACCG	*Xba*I
P2ProSmoR	GGATCCGAAGGCGTCGCGCTCGG	*BamH*I
P1ExpSmoR	CATATGAGCGATCTGGTGCAGGCG	*Nde*I
P2ExpSmoR	CTCGAGTCAGTCTTCGATCTCGCCT	*Xho*I
PT7up	TAATACGACTCACTATAGGG	–
PT7dw	GCTAGTTATTGCTCAGCGG	–

*E. coli, S. maltophilia* and *P. aeruginosa* strains were routinely grown in Luria-Bertani (LB) medium at 37°C. *A. tumefaciens* KYC55 was grown in AT medium (Fuqua and Winans, 1994) at 30°C. When required, the antibiotics were supplemented as follows: ampicillin (Ap) 20 μg/ml (*E. coli*); tetracycline (Tc) 17 μg/ml (*E. coli* and *P. aeruginosa*) or 2 μg/ml (*A. tumefaciens*); erythromycin (Erm) 50 μg/ml (*E. coli*) or 500 μg/ml (*S. maltophilia*); gentamicin (Gm) 10 μg/ml (*E. coli*), 100 μg/ml (*A. tumefaciens*) or 40 μg/ml (*S. maltophilia*); and spectinomycin (Spc) 100 μg/ml (*E. coli* and *A. tumefaciens*).

### Sequence determination and in silico analysis

A 5.5 kb fragment containing the ORFs of *smlt1840* and *smlt1839* plus their flanking regions was amplified from *S. maltophilia* strain E77 genomic DNA and subsequently sequenced (Macrogen). The sequence has been used for sequence alignments as well as reference to generate a Δ*smoR* mutant in this model strain. The fragment corresponding to *hchA-smoR* operon and its predicted promoter (1658 bp) was submitted to Genbank under the accession number KP691985. Annotation was done using BLAST (Altschul et al., 1990) and intergenic regions were manually inspected for palindromic motifs and *cis* elements that participate in regulating translation. Program RSAT (Thomas-Chollier et al., 2011) was used to scan for a pattern (the palindromic box) within all ORF upstream regions in the K279a genome. A simple screen for *lux*R-like genes using a *S. maltophilia* K279a genomic sequence (AM743169.1) was done by using BLAST and PSI-BLAST (Altschul et al., 1997) to detect remote homologs. Sequences of the *hchA-smoR* operon from other *S. maltophilia* strains were retrieved from their genome sequences at NCBI (http://www.ncbi.nlm.nih.gov/genome/). Translation of ORFs to amino-acid sequences and sequence alignments were done with MEGA 6 (Tamura et al., 2013) and then analyzed with SMART (Letunic et al., 2009) for the identification and annotation of protein domains. Nucleotide and protein sequences were aligned using the ClustalW module implemented in MEGA 6 and manually edited and visualized with BioEdit. Software was run with default parameters unless otherwise stated. Identification of “LuxR-like regulators chaperone HchA associated” in other Proteobacteria was predicted by InterPro (http://www.ebi.ac.uk/interpro/) (Mitchell et al., 2014).

### Preparation of fusion and expression vectors

Oligonucleotides used as primers and plasmids used in this study are listed in Tables 2 and 3, respectively. The transcriptional fusion construct for the *smoR* promoter in pBBR1MCS-5-*lacZ* (Fried et al., 2012) was generated by amplifying a fragment of 415 bp containing the putative promoter of the operon *hchA-smoR* (*smlt1840*-*smlt1839*) from *S. maltophilia* E77, using primers P1ProSmoR and P2ProSmoR and FastStart DNA polymerase (Roche). The fragment was digested using *Xba*I and *BamH*I and cloned into their respective restriction sites into pBBR5MCS-5-*lacZ*, generating pBBR5MCS-*PsmoR::LacZ*. This vector was electroporated (Choi et al., 2006) into *S. maltophilia* E77 and transformants were seeded onto LB plates supplemented with 40 μg/ml Gm.

**Table 2:** **Plasmids used in this study**.

**Plasmid**	**Relevant Characteristics**	**Source**
pGEM-Erm	Cloning vector carrying *Erm* resistance gene, *Amp^r^, Erm^r^*	This work
pEX18Tc	Suicide allelic exchange vector; *Tc^r^*	Hoang et al., 1998
pEXsmoR	pEX18Tc carrying E77 *smoR* flanking regions interrupted with *Erm* resistance gene, *Tc^r^, Erm^r^*	This work
pBBR1MCS-5	Broad-host-range cloning vector, *Gm^r^*	Kovach et al., 1995
pET22b	IPTG inducible expression vector, *Amp^r^*	Novagen
pET22b-smoR	IPTG inducible expression vector carrying *smoR* ORF, *Amp^r^*	This work
pBBR1MCS-5-*lacZ*	pBBR1MCS-5 plasmid carrying promoterless *lacZ* gene, *Gm^r^*	Fried et al., 2012
pBBR1MCS-5-*PsmoR::lacZ*	pBBR1MCS-5 plasmid carrying fusion *PsmoR::lacZ* gene, *Gm^r^*	This work
pME6000	Broad-host-range cloning vector, *Tc^r^*	Maurhofer et al., 1998
pME*Plac::aiiA*	pME6000 carrying lactonase *aiiA* gene from *B. subtilis* under the control of *Plac* promoter, *Tc^r^*	Reimmann et al., 2002

**Table 3:** **Strains used in this study**.

**Strains**	**Relevant characteristics**	**References**
*S. maltophilia*		
E77	Wild type	Ferrer-Navarro et al., 2013
E77 Δ*smoR*	E77 Δ*smoR* (Δ*smlt1839*), Erm*^r^*	This work
E77 pBBR1MCS-5-*lacZ*	E77 harboring vector pBBR1MCS-5-*lacZ*, Gm*^r^*	This work
E77 pBBR1MCS-5-*PsmoR::lacZ*	E77 harboring vector pBBR1MCS-5-*PsmoR::lacZ*, Gm*^r^*,	This work
E77 Δ*smoR* pBBR1MCS-5-*lacZ*	E77 Δ*smoR* harboring vector pBBR1MCS-5-*lacZ*, Gm*^r^*, Erm*^r^*	This work
E77 Δ*smoR* pBBR1MCS-5-*PsmoR::lacZ*	E77 Δ*smoR* harboring vector pBBR1MCS-5-*PsmoR::lacZ*, Gm*^r^*, Erm*^r^*	This work
*E. coli*		
DH5α	*recA1 endA1 hsdR17 gyrA96 supE44 thi-1 relA1*Δ(*lacZYA-argF)U169 deoR* Φ*80dlacZ*Δ*M15*	Lab. Collection
DH5α pEX*smoR*	DH5α harboring vector pEX*smoR*, Tc*^r^*, Erm*^r^*	This work
BL21 (DE3)	*fhuA2 [lon] ompT gal (λ DE3) [dcm] ΔhsdSλ DE3 = λ sBamHIo ΔEcoRI-B int::(lacI::PlacUV5::T7 gene1) i21 Δnin5*	Novagen
BL21 (DE3) pET22b	BL21 (DE3) harboring pET22b, Amp*^r^*	This work
BL21 (DE3) pET22b-*smoR*	BL21 (DE3) harboring pET22b-*smoR*, Amp*^r^*	This work
*P. aeruginosa*		
MPAO1	Wild type	Jacobs et al., 2003
MPAO1 pME600	MPAO1 harboring pME600, Tc*^r^*	
MPAO1 pME*Plac::aiiA*	MPAO1 harboring pME-*Plac::aiiA*, Tc*^r^*	This work
*A. tumefaciens*		
KYC55	KYC55 harboring vectors pJZ384, pJZ410 and pJZ372 Spc*^r^*, Gm*^r^*, Tc*^r^*	Zhu et al., 2003

To generate the expression vector for SmoR production in *E. coli*, the ORF of *smlt1839* was amplified using primer pair P1ExpSmoR-P2ExpSmoR and the amplified fragment was digested with *Nde*I and *Xho*I and cloned into their respective restriction sites into pET22b (Novagen), creating pET22b-*smoR. E. coli* strain BL21 (DE3) was transformed (Sambrook et al., 1989) with plasmid pET22b-*smoR* and transformants were seeded onto LB plates containing 20 μg/ml Amp.

The vectors pME6000 (Maurhofer et al., 1998) and pME*lacZ::aiiA* (Reimmann et al., 2002)—the latter carrying a transcriptional fusion between *lacZ* promoter and the ORF of the lactonase AiiA from *Bacillus subtilis* strain A24 (Dong et al., 2000)—were provided by the authors and were used to investigate the effect of the lactonase AiiA on the degradation of the AHL signals from *P. aeruginosa*. Both vectors were electroporated (Choi et al., 2006) into *P. aeruginosa* MPAO1 and transformants were seeded onto LB plates containing 17 μg/ml Tc.

### Generation of Δ*smoR* mutant

*S. maltophilia* E77 Δ*smoR* mutant was obtained by allelic-exchange recombination using erythromycin as antibiotic-resistance cassette. Briefly, *smoR* upstream and downstream flanking regions (993 and 863 bp, respectively) were amplified by PCR using primer pairs P1MutSmoR-P2MutSmoR (upstream region) and P3MutSmoR-P4MutSmoR (downstream region) and inserted, flanking an erythromycin cassette, into the suicide vector pEX18Tc (Hoang et al., 1998), generating plasmid pEX*smoR*. The erythromycin cassette was previously amplified from plasmid pGEM-Erm (Table 2) using primers PErm5′ and PErm3′. *S. maltophilia* E77 was electroporated (Choi et al., 2006) with the suicide vector pEX*smoR* and transformants were seeded onto LB plates containing 500 μg/mL Erm and subsequently streaked onto LB plates containing 17 μg/mL Tc to discard single cross-over events. *smoR* deletion was also verified by PCR using primer combinations P1DemSmoR-PErm5′rev (for upstream region) and P2DemSmoR-PErm3′rev (for downstream region). The obtained fragments were subsequently verified by sequencing (Macrogen).

### Measuring ß-galactosidase activity

To evaluate the expression levels of *hchA-smoR* promoter, ß-galactosidase assays were performed for the strains E77 wild type and Δ*smoR* mutant harboring either the vectors pBBR1MCS-5-*PsmoR:lacZ* or pBBR1MCS-5-*lacZ*—the latter used as a control– during growth in LB medium at 30°C, following the protocol described by Miller (1972). All bacterial cultures were started with an initial inoculum corresponding to an optical density at 550 nm (OD_550_) of 0.05. To determine the activity of the *hchA-smoR* promoter during growth curve, 0.1 ml-samples were taken at different times from 4 to 48 h. To investigate the effect of the presence of AHL molecules in the activity of the *hchA-smoR* promoter, initial cultures were supplemented with various synthetic AHLs (Cayman Chemical) –C6-HSL, oxo-C8-HSL, C8-HSL, and C10-HSL– with different concentrations (1 up to 10 μM), and 0.1 ml samples were taken and measured after 24 h and 48 h of incubation at 30°C. After analyzing the data we determined β-galactosidase specific activities in Miller Units (Miller, 1972). All AHL stocks were solubilized in 70% acetonitrile/water acidified with 0.1 M HCl final concentration. All experiments were performed by triplicate and comparison of ß-galactosidae activity was performed by One-Way analysis of variance (ANOVA) with a Bonferroni's multiple comparison post-test.

### Extraction, thin layer chromatography and bioassay of AHLs

To evaluate AHL produced by *P. aeruginosa*, 150 ml culture supernatants of strain MPAO1, or MPAO1 transformed with either pME6000 or pME*lacZ::aiiA* grown in LB at 37°C for 24 h (OD_550_ of about 2), were extracted with 300 ml of acidified ethyl acetate (0.1% acetic acid). The organic phase was evaporated to dryness using a rotary evaporator, and the residues were dissolved in an appropriate volume of acidified ethyl acetate. 5 μl aliquots of dissolved ethyl acetate residues were spotted onto C18 reverse-phase plate (Merck) (Shaw et al., 1997) and separated with methanol:water (60:40, vol/vol) as running solvent. TLC plates were subsequently air-dried for at least 1 h and overlaid with 100 ml of unsolidified warm AT medium containing 0.8% agar, 60 μg/ml X-Gal and the AHL reporter strain KYC55 to an OD_550_ of ca. 0.8. TLC plates were incubated overnight at 30°C, and AHL activity was identified by the presence of blue spots. 2 μl of the aforementioned synthetic AHLs were also tested in TLC coupled to bioassay and used as a control.

### AHL binding assay

The AHL binding assay was performed as described (Subramoni and Venturi, 2009b), with few modifications. 20 ml cultures of *E. coli* BL21 (DE3) harboring either pET22b or pET22b-*smoR* were grown at 37°C in LB medium containing 10 μg/ml Amp to an OD_550_ of 0.1. Bacterial cultures were then supplemented with different AHL molecules (C6-HSL, oxo-C8-HSL, C8-HSL and C10-HSL) at 10 and 20 μM final concentration and cultures were incubated until reaching an OD_550_ of 0.6. SmoR production was induced with 1 μM final concentration of IPTG and the cultures were additionally incubated for 3.5 h. OD_550_ was measured and the cultures were adjusted to contain an equal number of cells per mL and subsequently centrifuged. Cell pellets were washed three times with 10 ml of PBS and cellular suspensions were extracted twice with the same volume of acidified ethyl acetate. The extracts were then dried, dissolved in ethyl acetate and analyzed by TLC coupled to AHL bioassay, as described above. An aliquot of the corresponding induced culture was previously removed to control the identity of the overproduced recombinant protein (see Supplementary Figure S1).

### Swarming assay

Swarming motility was assayed on BM2 medium plates (62 mM potassium phosphate buffer, pH = 7, 2 mM MgSO_4_, 10 μM FeSO_4_, 0.5% [wt/vol] casamino acids, supplemented with glucose 0.4% and solidified with 0.5% BD Difco Noble agar) (Overhage et al., 2007). Plates containing 20 ml of fresh swarm medium were dried under a laminar-flow hood for 20 min before pin-inoculation. When indicated, solidified swarm plates were supplemented with 10 μl of concentrated culture supernatant—extracted as described above– of *P. aeruginosa* MPAO1 and its derivative strains, as indicated in figure captions. Inoculated swarm plates were sealed to maintain the humidity and incubated at 30°C up to five days. Swarming experiments were done in triplicate and representative images are shown.

## Results

### Smlt1839 contains both the AHL- and DNA-binding LuxR domains

**Figure 1:**
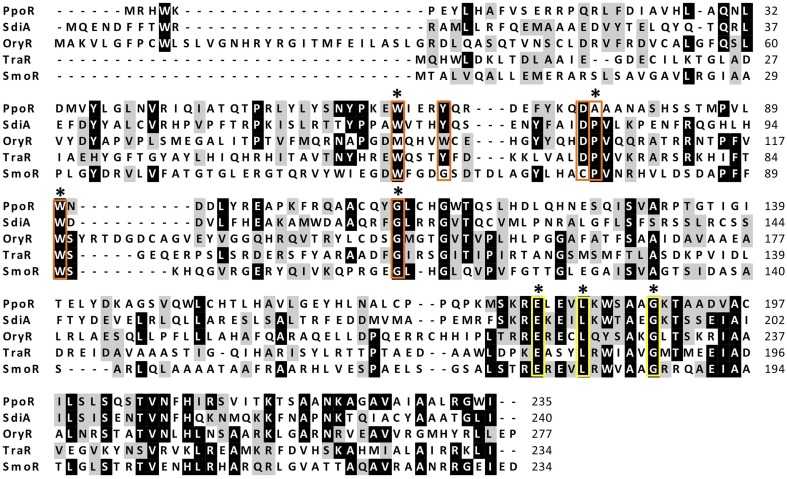
**Protein alignment of orthologs LuxR solos from diverse Proteobacteria; PpoR (FM992078): *Pseudomonas putida* strain RD8MR3; SdiA (AAC08299.1): *Salmonella enterica* subsp. *enterica* serovar Typhimurium strain LT2; OryR (AAR91700.1): *Xanthomonas oryzae* pv. *oryzae*; TraR (AAZ50597.1): *A. tumefaciens*; SmoR: *S. maltophilia* E77 (KP691985)**. Red boxes highlight amino acids implicated in AHL binding and yellow boxes indicate residues involved in DNA binding (HTH). From these, conserved amino acids in E77 are marked with an asterisk.

It is known that certain non AHL-producing bacteria are able to sense AHLs and regulate various biological functions in response to signals produced by others through diverse LuxR-like regulators (Patankar and González, 2009). The genome of *S. maltophilia* strain K279a (Crossman et al., 2008) was revisited for the presence of genes encoding putative LuxR-like regulators. Besides the eight genes already annotated as two-component-system response regulators of the LuxR family, a total of seven additional hypothetical LuxR regulators were identified (Table 4), none of them associated to a *luxI* homolog. All these LuxR-solo candidates were examined in detail for the presence of the typical N-terminal AHL-binding domain (PFAM 03472) and the C-terminal helix-turn-helix (HTH) DNA-binding domain (PFAM 00196) (Miller and Bassler, 2001). From these, only the gene *smlt1839* was found to encode for a protein—here named SmoR (*Stenotrophomonas maltophilia* orphan regulator)—containing both conserved domains (Table 4). A subsequent protein alignment with known orphan regulators from distinct Proteobacteria including PpoR from *P. putida*, SdiA from *S. enterica*, OryR from *X*. *oryzae* pv. *oryzae*, and TraR from *A. tumefaciens*, revealed that at the N-terminal domain four out of six residues involved in AHL binding (Patankar and González, 2009) are conserved in SmoR (Figure 1). Concerning the C-terminal HTH domain, the three residues responsible for DNA binding (Hanzelka and Greenberg, 1995; Fuqua et al., 1996) are also conserved in SmoR (Figure 1). Further protein BLAST analysis revealed that SmoR is largely conserved among *S. maltophilia* (data not shown). These results suggest that the conserved regulator SmoR (Smlt1839) could be implicated in signaling systems in *S. maltophilia*.

**Table 4:** **Hypothetical LuxR-like regulators annotated in the genome of *S. maltophilia* strain K279a**.

**Locus ID**	**Lenght**	**N-ter Domain**	**C-ter Domain**	**Annotation in K279a**
Smlt1839	234	AHL	LuxR HTH	LuxR family transcriptional regulator
Smlt0195	212	REC	LuxR HTH	LuxR family two component response regulator
Smlt0389	223	REC	LuxR HTH	Two component transcriptional regulator, LuxR family
Smlt2299	210	REC	LuxR HTH	Response regulator protein LuxR family
Smlt2366	208	REC	LuxR HTH	Two-component response regulator, LuxR family
Smlt4224	212	REC	LuxR HTH	LuxR family two-component response regulator
Smlt0367	200	REC	LuxR HTH	Two-component system response regulator, LuxR family
Smlt0400	254	REC	LuxR HTH	Two-component response regulator transcriptional regulator
Smlt0881	213	REC	LuxR HTH	Two-component response regulator transcriptional regulator
Smlt1255	213	REC	LuxR HTH	Two-component response regulator transcriptional regulator
Smlt1788	215	REC	LuxR HTH	Two-component response regulator transcriptional regulator
Smlt2595	224	REC	LuxR HTH	Two-component response regulator transcriptional regulator
Smlt2658	213	REC	LuxR HTH	Two-component response regulator transcriptional regulator
Smlt2891	217	REC	LuxR HTH	Two-component response regulator transcriptional regulator
Smlt4624	221	REC	LuxR HTH	Two component system response regulator

### *S. maltophilia* SmoR binds AHLs

It has been demonstrated that various LuxR solos containing the AHL-binding domain are able to bind to one or more AHL signal molecules. To determine whether in *S. maltophilia* the regulator SmoR could bind to any of these signals, an AHL-binding assay was performed. The appropriate overexpression of *S. maltophilia smoR* in *E. coli* strain BL21 (DE3) was validated by MALDI-MS analysis prior to initiate the AHL-binding assay (see Supplementary Figure S1).

**Figure 2:**
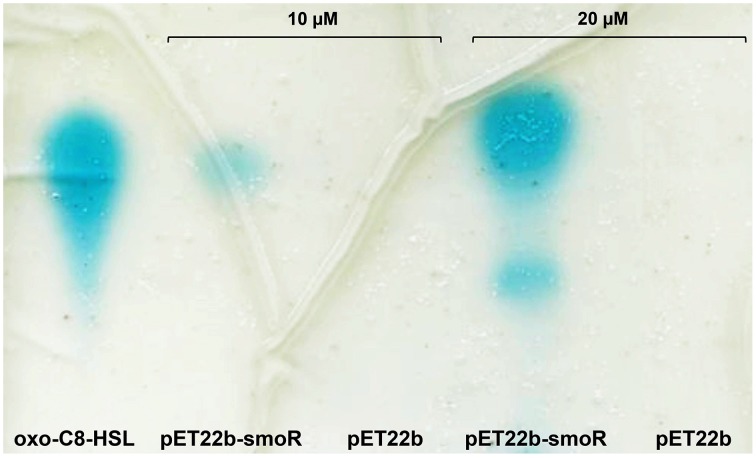
**Representative image of AHL binding assay done for *E. coli* BL21 (DE3) harboring the expression vector pET22b-smoR or the empty vector pET22b grown in LB supplemented with AHLs at concentration of either 10 or 20 μM**. Blue spots correspond to AHL signal. oxo-C8-HSL was used as control.

*E. coli* BL21 (DE3) harboring either the empty vector pET-22b or the one overproducing SmoR were grown in a rich medium supplemented with a variety of AHLs (see Materials and Methods). After the appropriate incubation time, the culture supernatant was removed and the cell pellet was washed and subsequently extracted with acidified ethyl acetate. Concentrated cell extracts were visualized by TLC coupled to the AHL bioassay, resulting in the detection of the signal oxo-C8-HSL (Figure 2). Likewise, it was observed that the detection depends on AHL concentration, since the culture supplemented with 20 μM oxo-C8-HSL presented a more intense spot compared to that supplemented with 10 μM. On the other hand, cell extracts from *E. coli* cells containing the empty vector did not show detectable AHL activity (Figure 2). The experiment was performed by triplicate and non-systematic binding was observed for the other AHLs tested. Overall, this indicates that *S. maltophilia* could sense AHL signal molecules –in particular oxo-C8-HSL– through the LuxR solo SmoR.

### *hchA* and *smoR* are part of the same operon, and operon expression is growth-phase- and AHL-dependent

In *S. maltophilia* K279a, the gene encoding the regulator SmoR is localized downstream of the gene encoding for the chaperone HchA (Smlt1840), separated by only five nucleotides, indicating that both genes could form the operon *hchA-smoR*. This genetic organization is conserved in all available *S. maltophilia* genomes and also in the clinical strain E77 used in the present study. The upstream genes to *hchA* in strains D457 and JV3 are oriented in the opposite direction, indicating that there must be a promoter preceding *hchA* and confirming the existence of a bicistronic operon. Interestingly, this operon has been observed only in few Gammaproteobacteria, including *Pseudomonas* spp., *Vibrio* spp., *Acinetobacter* spp., *Serratia* spp., among few others, as predicted by InterPro (Family IPR019941). Accordingly, these regulator elements are annotated as “LuxR chaperone HchA-associated”. In *E. coli*, the gene *hchA* encodes for the chaperone Hsp31, which displays a high protein identity (60%) to *S. maltophilia* HchA. However, *E. coli hchA* is an isolated gene in this bacterial genome. It has been shown that Hsp31 is a glyoxalase that plays a central role in detoxification of dicarbonyl radicals (Subedi et al., 2011) as well as in the response to bacterial stress such as heat shock (Mujacic et al., 2004; Mujacic and Baneyx, 2006) and acidic conditions (Mujacic and Baneyx, 2007). In these mentioned studies it has been reported that transcription of *hchA* is induced at high population density.

**Figure 3:**
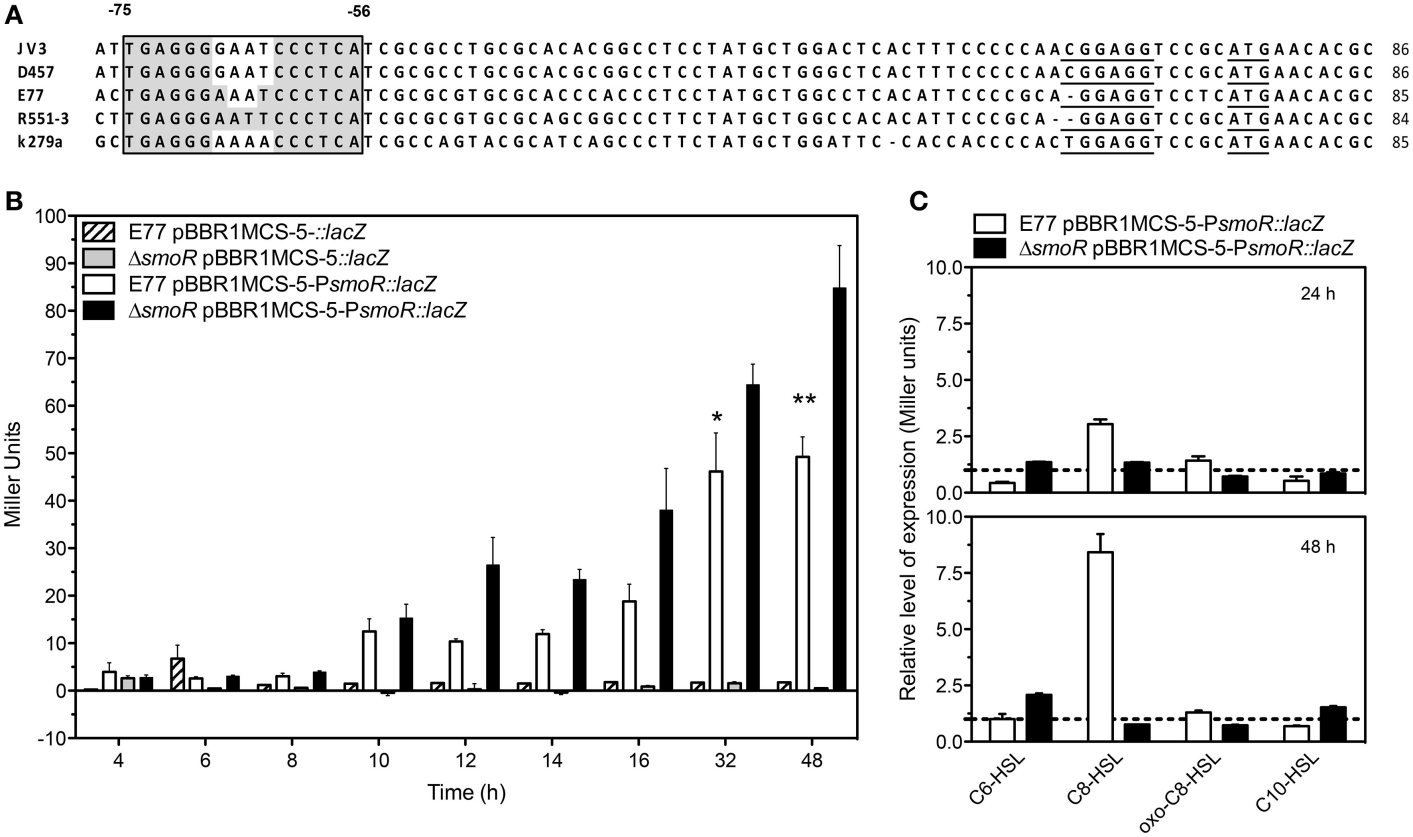
**(A)** Alignment of the *hchA-smoR* promoter from different *S. maltophilia* strains. Gray shadow indicates the palindromic motif. Underlined sequences correspond to the putative ribosome binding site (RBS) and the start codon (ATG). **(B)** ß-galactosidase assay expressed in Miller Units of E77 WT and Δ*smoR* mutant both harboring either pBBR1MCS-5*::lacZ* or pBBR1MCS-5-*PsmoR::lacZ* monitored during 48 h. Under these conditions all tested strains showed similar growth curves. **(C)** ß-galactosidase activity of E77 wild type and Δ*smoR* mutant both harboring vector pBBR1MCS-5-*PsmoR::lacZ* grown in LB supplemented with diverse AHLs at 1 μM concentration each and incubated at 30°C for 24 or 48 h. Relative expression values are reported as Miller units of β-galactosidase activity in cells grown under the indicated growth conditions divided by the values in cells grown in LB broth without AHLs. ^*^P < 0.05; ^**^p < 0.01.

In *S. maltophilia* E77 the upstream *hchA-smoR* operon region was examined for the presence of a putative promoter. Despite canonical core promoter elements are not found in this region, DNA sequence alignment of this region from different *S. maltophilia* strains revealed a conserved ribosome-binding site and a palindromic conserved motif spanning from positions −75 to −56 with respect to the translational start site (Figure 3A). The genome of *S. maltophilia* K279a was also evaluated for the presence of this palindromic motif in other promoter gene regions. Curiously, only a highly similar box (68.75% identity) was found in the intergenic region between *smlt2137* (position −143 to −159) and *smlt2138* (position −15 to −31). Those genes encode for a universal stress-family protein (Smlt2137) and for the transcriptional regulator NfxB (Smlt2138) (Shiba et al., 1995), respectively.

The *hchA-smoR* upstream region including the palindromic sequence motif was fused to *lacZ* gene and used in ß-galactosidase experiments to corroborate the existence of a promoter. The expression levels of the fusion construct *PsmoR::lacZ* were monitored in *S. maltophilia* strain E77 wild type and its derivative Δ*smoR* mutant, in order to determine whether the expression pattern of the operon was similar to that previously described for the *hchA* gene in *E. coli* (Mujacic and Baneyx, 2006, 2007). Additionally, the role of SmoR in such expression was also evaluated, since the autoregulation of LuxR proteins is relatively common (Shadel and Baldwin, 1992; Chatterjee et al., 1996; Minogue et al., 2002).

The results from the ß-galactosidase experiments indicate that the promoter activity is slight at low cell densities and increases with bacterial-growth rate, showing a maximum at 48 h (stationary phase, Figure 3B). Although this tendency was observed in both wild type and Δ*smoR* backgrounds, the absence of SmoR led to increased levels of expression compared to the wild type strain E77, specially at late stationary phase of growth (Figure 3B) (*P* < 0.01). Under these standard conditions (LB and 30°C) all tested strains showed similar growth curves reaching stationary phase by 24 h approximately. These results suggest that operon components would act at high cell densities, participating likely in this sort of stress response as observed for Hsp31 in *E. coli*.

Since we observed *in vitro* that SmoR bind the signal oxo-C8-HSL (Figure 2) and it is known that some active LuxR-like proteins can autoregulate their own expression, we wanted to investigate the role of SmoR on the regulation of *hchA-smoR* operon expression in the presence of AHLs. To do that, β-galactosidase assays were performed for the same strains supplemented with various AHLs (see Materials and Methods). The results showed that the expression of the operon was also modulated by the presence of these signal molecules. In particular, supplementation of E77 wild type with 1 μM C8-HSL resulted into approximately threefold and 8-fold operon activation at 24 and 48 h post induction respectively, compared to the expression levels of the supplemented Δ*smoR* mutant (Figure 3C). This indicates that SmoR is involved in AHL-dependent operon-expression regulation.

### AHLs produced by *P. aeruginosa* promote swarming motility in *S. maltophilia*, SmoR playing a central role in this stimulation

It is known that AHL signals modulate several biological functions not only in AHL-producing bacteria, but also in certain species lacking typical *luxI/luxR* systems which are still able to respond to exogenous AHLs through diverse orphan LuxR. One of the behaviors that have raised more interest recently and is commonly regulated by AHL-QS is swarming motility (Daniels et al., 2004). Swarming motility is a rapid and coordinated translocation of a bacterial population across semi-solid surfaces, which frequently requires quorum sensing-mediated synchronization (Kearns, 2010).

**Figure 4:**
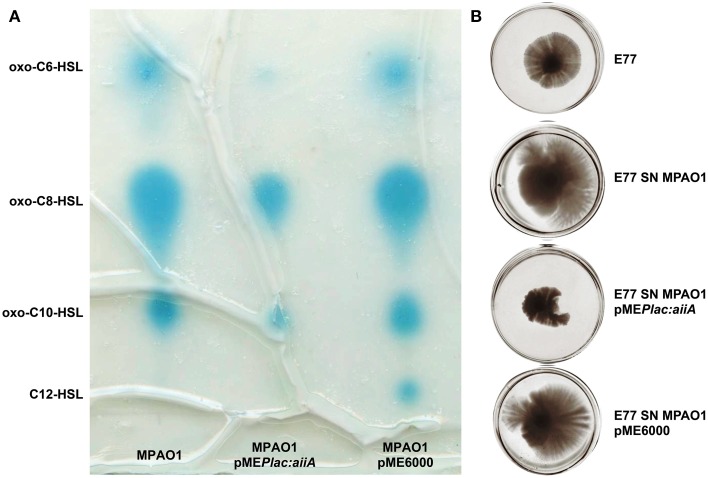
**Representative images of (A) TLC coupled to AHL bioassay of concentrated supernatants of *P. aeruginosa* MPAO1, MPAO1 pME*Plac::aiiA*, and MPAO1 pME6000**. Blue spots correspond to AHL molecules. **(B)** Swarming motility in BM2 0.5% agar medium plates of *S. maltophilia* E77 supplemented with 10 μl of the same concentrated supernatants (SN) and incubated at 30°C for 5 days.

Since *S. maltophilia* frequently cohabit with AHL-producing bacteria –i.e., *P. aeruginosa* (Moskowitz et al., 2005)– we investigated the effect that *P. aeruginosa* AHLs could have on *S. maltophilia* regulation, more specifically on swarming motility. To that end, we supplemented *S. maltophilia* swarming plates with concentrated supernatants of *P. aeruginosa* strain MPAO1 wild type and MPAO1 harboring the plasmid pME*Plac::aiiA*, which expresses the lactonase AiiA from *B. subtilis* (Dong et al., 2000). In order to corroborate the effect of the lactonase AiiA in AHL degradation, TLC followed by AHL bioassay was performed in parallel to swarming assays. As shown in Figure 4, expression of AiiA led to a decrease of *P. aeruginosa* AHL production and, interestingly, it resulted in a drastic reduction of *S. maltophilia* swarming stimulation. These results would indicate that AHL signals produced by *P. aeruginosa* promote swarming motility in *S. maltophilia*.

**Figure 5:**
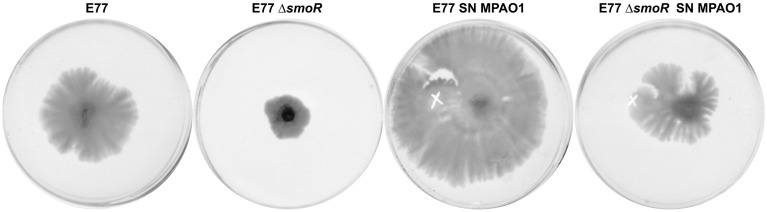
**Representative images of swarming motility in BM2 0.5% agar medium plates of *S. maltophilia* E77 WT and Δ*smoR* mutant supplemented with 10 μl of concentrated supernatants (SN) of *P. aeruginosa* strain MPAO1 WT and incubated at 30°C for 5 days**. The white cross indicates the zone of the addition of the concentrated supernatants.

Several evidences drive us to suggest that SmoR was responsible for the observed AHL-mediated swarming stimulation in *S. maltophilia* strain E77. It has been shown that, in the related bacteria *X. oryzae* pv. *oryzae*, the LuxR solo OryR also regulates certain motility processes, including swarming (González et al., 2013). In order to test such hypothesis in *S. maltophilia*, MPAO1 concentrated supernatants were spotted onto swarming plates seeded with the strains E77 wild type and the Δ*smoR* mutant. The results further demonstrate that, as observed before, the MPAO1 concentrated supernatant significantly promotes the swarming ability of E77 (Figure 5). To the contrary, mutation of *smoR* resulted into a loss of swarming motility and supplementation with *P. aeruginosa* concentrated supernatant produced only a minor stimulation compared to E77 WT, perhaps due to the surfactant character of the AHL signals and other hydrophobic molecules present in its supernatant. These results strongly suggest that AHLs produced exogenously promote swarming motility in *S. maltophilia* and SmoR plays a central role in such stimulation (Figure 5).

## Discussion

The interest in LuxR-solo investigation has grown among microbiologists, since these regulatory elements may unveil novel signaling systems. The increment of public sequenced genomes has permitted scientists to screen an extraordinary number of bacterial genomes and identify new regulator elements. It recently has been shown in bacteria that 75% of the annotated *luxR*-type genes encode for a “LuxR solo” (Hudaiberdiev et al., 2015), which is a surprisingly high proportion. To date, it is known that various LuxR can regulate a range of biological function in response to: endogenous AHLs (Chugani et al., 2001; Lequette et al., 2006); exogenous AHLs produced by neighboring bacteria (Ahmer et al., 1998; Michael et al., 2001; Ahmer, 2004; Yao et al., 2006); the novel bacterial signaling molecules dialkylresorcinols (DARs) and cyclohexanediones (CHDs) (Brameyer et al., 2015); or even signals produced by plants (Ferluga et al., 2007; Ferluga and Venturi, 2009). Considering the high heterogeneity and complexity of LuxR solos in bacteria, it is highly probable that new regulatory networks are there to discover.

The aim of the present study was to identify and investigate putative LuxR solos in *S. maltophilia*. After screening the genome of the model strain K279a (Crossman et al., 2008), only the protein encoded by the *smlt1839* gene (SmoR) was found to display the two typical LuxR domains: the autoinducer-binding domain (Shadel et al., 1990; Slock et al., 1990) and the DNA-binding helix-turn-helix (HTH) domain (Choi and Greenberg, 1991; Fuqua and Winans, 1994). Analysis at the amino-acid sequence level revealed that from the 9 crucial residues (Whitehead et al., 2001; Zhang et al., 2002), seven are conserved in SmoR. Diverse structural and functional analyses of several LuxR regulators have revealed that, while the DNA-binding domain (HTH) is widely conserved, the autoinducer-binding domain presents substantial variability, likely to adjust to a range of signal molecules (Vannini et al., 2002; Zhang et al., 2002; Yao et al., 2006; Bottomley et al., 2007). This is the situation observed in *S. maltophilia* SmoR, where the AHL-binding domain presents substitutions in residues 62 and 74, while the HTH domain is perfectly conserved (Figure 1). It has been reported that in *A. tumefaciens*, the LuxR solo TraR also binds to oxo-C8-HSL through a hydrophobic cavity composed of six conserved residues (Vannini et al., 2002; Zhang et al., 2002). Single-mutation analysis of some of these residues does not render AHL-binding incompetent TraR, but elevated signal concentrations—5000 to 10,000-fold– are then needed to achieve oxo-C8-HSL binding (Koch et al., 2005).

Additionally, it has been demonstrated that in *X. oryzae* pv. *oryzae* (*Xoo*), the LuxR solo OryR—which presents different critical amino acids in the AHL-binding domain– recognizes plant signals rather than AHLs (Ferluga et al., 2007; Ferluga and Venturi, 2009; González et al., 2013). Taking into account this observations and considering the variation in the AHL-binding domain of SmoR, it has been suggested that *S*. *maltophilia* SmoR might also recognize plant signals (Crossman et al., 2008), a possibility that has not been yet validated. We show here that SmoR binds AHLs, in particular the signal oxo-C8-HSL (Figure 2).

Genomic organization and sequence analysis have shown that *oryR* and *xccR* are not orthologous to *smoR*. Indeed, SmoR belongs to a recently classified subfamily designated “LuxR-like regulators chaperone HchA associated” as predicted by InterPro (http://www.ebi.ac.uk/interpro/) (Mitchell et al., 2014). Curiously, most bacteria sharing this particular operon display AHL production. However, there has been no functional study on the *hchA*-*smoR* operon. Nevertheless, the high homology observed between *S. maltophilia* HchA and *E. coli* Hsp31 suggests that, apart from being orthologous genes, both HchA and Hsp31 may regulate similar functions. In the genome of *E. coli*, the *hchA* gene is monocistronic. Its gene product Hsp31 has been widely studied in *E. coli* and its diverse functions have been elucidated. Initially, Hsp31 was reported to be a heat-inducible chaperone, showing feeble protease activity (Sastry et al., 2002; Malki et al., 2003, 2005). Further, it was shown that Hsp31 participates in heat shock and starvation response (Mujacic et al., 2004; Mujacic and Baneyx, 2006) as well as resistance to acid environments, generated during the stationary phase of growth (Mujacic and Baneyx, 2007). Recently it has been demonstrated that Hsp31 is a glyoxalase that participates in the detoxification of dicarbonyl stress (Subedi et al., 2011), which spans even more the functional spectrum of this versatile protein. Overall, this protein appears to act in front of stress environments derived from high cell density, a situation where QS systems are also active. In this line, we have shown that in *S. maltophilia* also a high cell density induces the expression of the *hchA*-*smoR* operon (Figure 3B). This suggests that *S. maltophilia* HchA may also act in front of stress situations such as starvation or accumulation of secondary metabolites, as described for *E. coli* Hsp31 (Mujacic and Baneyx, 2006, 2007; Subedi et al., 2011). In addition, we have observed that mutation of *smoR* results in a higher activity of operon during growth, which suggests that SmoR may act as a repressor in this situation (Figure 3B). On the other hand, we have observed in the wild type background that the presence of the exogenous signal C8-HSL significantly enhance *in vivo* promoter activity (Figure 3C). Although no systematic binding was observed for C8-HSL in the AHL-binding assay (data not shown), ß-galactosidase experiments make us reconsider whether SmoR could also bind to this signal, apart from oxo-C8-HSL. A possible explanation could rely on the use of different systems in each technique (note that AHL-binding assay was done in *E. coli*, while ß-galactosidase experiments have been performed in *S. maltophilia*). Therefore, further studies will be necessary to better understand the role of both eight-carbon AHL in the SmoR-dependent regulation. Interestingly, we have observed that the LuxR solos QscR (PA1898) from *P. aeruginosa* and the SmoR are putative orthologs. A new *in vivo* mechanism for LuxR activation depending on the free and signal-bond state has been proposed for QscR (Oinuma and Greenberg, 2011). It has been reported that although QscR binding to the signal 3-oxo-C6-HSL has a slightly effect on its own transcription (Lee et al., 2006), it can stimulate dimerization and activation, while preventing it from proteolysis (Oinuma and Greenberg, 2011; Chugani and Greenberg, 2014). A similar mechanism have been described for TraR regulator, suggesting that this new mechanisms might be widespread among LuxR homologs (Zhu and Winans, 1999, 2001), perhaps including SmoR. Altogether, we hypothesize that the regulator SmoR might bind to the palindromic box and block operon expression, until the presence of signal molecules bind to SmoR and avoid operon repression. Curiously, a highly related palindromic box was also identified between genes *smlt2137* (universal stress family protein) and *smlt2138* (transcriptional regulator *nfxB*) (Mitchell et al., 2014), two proteins that appear to be also involved in stress response. Nevertheless, determining whether SmoR could play an activator or a repressor role as well as which other genes could be under its regulation is something that will require further studies. Overall, our results suggest that in *S. maltophilia*, the *hchA-smoR* operon could have two important functions: (i) detoxification and recycling of secondary metabolites (HchA) and (ii) response to QS signals (SmoR), both deriving from situations of high cell density.

Interactions within microbial populations are common and essential for community maintenance (Ryan and Dow, 2008). *In vitro* studies of microbial consortia represent a first approach to uncover the complex interaction networks that occur between organisms in nature. Interspecific communication between *S. maltophilia* and *P. aeruginosa* has been a subject of investigation in the last years, since these two bacterial species usually share ecological niches and, importantly, they are frequently co-isolated in lungs of cystic fibrosis (CF) patients (Moskowitz et al., 2005). It has been reported that the DSF signal produced by *S. maltophilia* modulates *P. aeruginosa* behavior, including biofilm development and polymixin tolerance (Ryan et al., 2008) as well as virulence and persistence in lungs of CF patients (Twomey et al., 2012). However, as far as we know, the communication between these two potential human pathogens has been only studied in a unidirectional-way. We report here for the first time that *S. maltophilia* can also respond to signaling molecules produced by *P. aeruginosa*. In particular, we have shown that AHLs produced by *P. aeruginosa* stimulate swarming motility in *S. maltophilia* (Figure 4), a process in which SmoR might play a central role (Figure 5). Nevertheless, we cannot exclude that some other molecules present in the *P. aeruginosa* supernatant, such as rhamnolipids (Caiazza et al., 2005), could also participate in swarming stimulation. If so, it would explain the slightly stimulation observed in the Δ*smoR* mutant, which perhaps could initiate its swarming motility due to the presence of rhamnolipids rather than AHL signals.

It is well established that complex population behaviors such as swarming motility are commonly regulated by AHL-QS systems (Daniels et al., 2004). Furthermore, previous studies have demonstrated that heterologous expression of lactonase AiiA from *B. subtilis* (Dong et al., 2000) reduces AHLs production and swarming motility in *P. aeruginosa* (Reimmann et al., 2002) and *Burkholderia cepacia* species (Wopperer et al., 2006). Regulation of bacterial motility by LuxR solos has been also reported. In *Xoo*, OryR positively regulates swimming and swarming motility by binding to the promoter of numerous flagella genes in response to plant signaling molecules (González et al., 2013).

To date, the only QS system described in *S*. *maltophilia* is DSF-QS, which is based on the signaling fatty acid molecule 11-cis-2-decenoic acid (Huang and Lee Wong, 2007; Huedo et al., 2014). Previous studies have evidenced that the DSF system regulates several virulence-related processes including bacterial motility, biofilm formation, antibiotic resistance and virulence (Fouhy et al., 2007; Deng et al., 2011; Huedo et al., 2014). In the related bacterium *Burkholderia cenocepacia*, both QS systems co-exist: the DSF –designated BDSF– (Boon et al., 2008; Deng et al., 2010) and the AHL (Wopperer et al., 2006) systems. Interestingly, it has been shown that certain biological functions such as motility, biofilm formation and virulence, are co-regulated by the BDSF- and AHL-dependent QS systems in *B. cenocepacia* (Deng et al., 2009, 2013). Although AHL production has not been reported in *S. maltophilia*, our findings suggest that, besides DSF, exogenous AHL signals could also regulate QS-related phenotypes—as observed for swarming motility–, by interacting with the LuxR solo SmoR.

### Conflict of interest statement

The authors declare that the research was conducted in the absence of any commercial or financial relationships that could be construed as a potential conflict of interest.

## References

[B1] Ahmer B. M. van Reeuwijk J. Timmers C. D. Valentine P. J. Heffron F. (1998). Salmonella typhimurium encodes an SdiA homolog, a putative quorum sensor of the LuxR family, that regulates genes on the virulence plasmid. J. Bacteriol. 180, 1185–1193. 9495757 10.1128/jb.180.5.1185-1193.1998 PMC107006

[B2] Ahmer B. M. M. (2004). Cell-to-cell signalling in *Escherichia coli* and Salmonella enterica. Mol. Microbiol. 52, 933–945. 10.1111/j.1365-2958.2004.04054.x 15130116

[B3] Altschul S. F. Gish W. Miller W. Myers E. W. Lipman D. J. (1990). Basic local alignment search tool. J. Mol. Biol. 215, 403–410. 10.1016/S0022-2836(05)80360-2 2231712

[B4] Altschul S. F. Madden T. L. Schäffer A. A. Zhang J. Zhang Z. Miller W.. (1997). Gapped BLAST and PSI-BLAST: a new generation of protein database search programs. Nucleic Acids Res. 25, 3389–3402. 10.1093/nar/25.17.3389 9254694 PMC146917

[B5] Boon C. Deng Y. Wang L.-H. He Y. Xu J.-L. Fan Y.. (2008). A novel DSF-like signal from Burkholderia cenocepacia interferes with Candida albicans morphological transition. ISME J. 2, 27–36. 10.1038/ismej.2007.76 18049456

[B6] Bottomley M. J. Muraglia E. Bazzo R. Carfì A. (2007). Molecular insights into quorum sensing in the human pathogen *Pseudomonas aeruginosa* from the structure of the virulence regulator LasR bound to its autoinducer. J. Biol. Chem. 282, 13592–13600. 10.1074/jbc.M700556200 17363368

[B7] Brameyer S. Kresovic D. Bode H. B. Heermann R. (2015). Dialkylresorcinols as bacterial signaling molecules. Proc. Natl. Acad. Sci. U.S.A. 112, 572–577. 10.1073/pnas.1417685112 25550519 PMC4299209

[B8] Brooke J. S. (2012). *Stenotrophomonas maltophilia*: an emerging global opportunistic pathogen. Clin. Microbiol. Rev. 25, 2–41. 10.1128/CMR.00019-11 22232370 PMC3255966

[B9] Caiazza N. C. Shanks R. M. Q. O'Toole G. A. (2005). Rhamnolipids modulate swarming motility patterns of *Pseudomonas aeruginosa*. J. Bacteriol. 187, 7351–7361. 10.1128/JB.187.21.7351-7361.2005 16237018 PMC1273001

[B10] Chatterjee J. Miyamoto C. M. Meighen E. A. (1996). Autoregulation of luxR: the Vibrio harveyi lux-operon activator functions as a repressor. Mol. Microbiol. 20, 415–425. 10.1111/j.1365-2958.1996.tb02628.x 8733239

[B11] Choi K.-H. Kumar A. Schweizer H. P. (2006). A 10-min method for preparation of highly electrocompetent *Pseudomonas aeruginosa* cells: application for DNA fragment transfer between chromosomes and plasmid transformation. J. Microbiol. Methods 64, 391–397. 10.1016/j.mimet.2005.06.001 15987659

[B12] Choi S. H. Greenberg E. P. (1991). The C-terminal region of the Vibrio fischeri LuxR protein contains an inducer-independent lux gene activating domain. Proc. Natl. Acad. Sci. U.S.A. 88, 11115–11119. 10.1073/pnas.88.24.11115 1763027 PMC53084

[B13] Chugani S. Greenberg E. P. (2014). An evolving perspective on the *Pseudomonas aeruginosa* orphan quorum sensing regulator QscR. Front. Cell. Infect. Microbiol. 452. 10.3389/fcimb.2014.00152 25389523 PMC4211393

[B14] Chugani S. A. Whiteley M. Lee K. M. D'Argenio D. Manoil C. Greenberg E. P. (2001). QscR, a modulator of quorum-sensing signal synthesis and virulence in *Pseudomonas aeruginosa*. Proc. Natl. Acad. Sci. U.S.A. 98, 2752–2757. 10.1073/pnas.051624298 11226312 PMC30211

[B15] Crossman L. C. Gould V. C. Dow J. M. Vernikos G. S. Okazaki A. Sebaihia M.. (2008). The complete genome, comparative and functional analysis of *Stenotrophomonas maltophilia* reveals an organism heavily shielded by drug resistance determinants. Genome Biol. 9:R74. 10.1186/gb-2008-9-4-r74 18419807 PMC2643945

[B16] Daniels R. Vanderleyden J. Michiels J. (2004). Quorum sensing and swarming migration in bacteria. FEMS Microbiol. Rev. 28, 261–289. 10.1016/j.femsre.2003.09.004 15449604

[B17] Demko C. A. Stern R. C. Doershuk C. F. (1998). *Stenotrophomonas maltophilia* in cystic fibrosis: incidence and prevalence. Pediatr. Pulmonol. 25, 304–308. 9635931 10.1002/(sici)1099-0496(199805)25:5<304::aid-ppul3>3.0.co;2-i

[B18] Deng Y. Boon C. Eberl L. Zhang L.-H. (2009). Differential modulation of *Burkholderia cenocepacia* virulence and energy metabolism by the quorum-sensing signal BDSF and its synthase. J. Bacteriol. 191, 7270–7278. 10.1128/JB.00681-09 19801414 PMC2786553

[B19] Deng Y. Lim A. Wang J. Zhou T. Chen S. Lee J.. (2013). Cis-2-dodecenoic acid quorum sensing system modulates N-acyl homoserine lactone production through RpfR and cyclic di-GMP turnover in Burkholderia cenocepacia. BMC Microbiol. 13:148. 10.1186/1471-2180-13-148 23815566 PMC3703271

[B20] Deng Y. Wu J. Eberl L. Zhang L.-H. (2010). Structural and functional characterization of diffusible signal factor family quorum-sensing signals produced by members of the Burkholderia cepacia complex. Appl. Environ. Microbiol. 76, 4675–4683. 10.1128/AEM.00480-10 20511428 PMC2901730

[B21] Deng Y. Wu J. Tao F. Zhang L.-H. (2011). Listening to a new language: DSF-based quorum sensing in Gram-negative bacteria. Chem. Rev. 111, 160–173. 10.1021/cr100354f 21166386

[B22] Devine J. H. Shadel G. S. Baldwin T. O. (1989). Identification of the operator of the lux regulon from the Vibrio fischeri strain ATCC7744. Proc. Natl. Acad. Sci. U.S.A. 86, 5688–5692. 10.1073/pnas.86.15.5688 2762291 PMC297695

[B23] Dong Y.-H. Xu J.-L. Li X.-Z. Zhang L.-H. (2000). AiiA, an enzyme that inactivates the acylhomoserine lactone quorum-sensing signal and attenuates the virulence of Erwinia carotovora. Proc. Natl. Acad. Sci. U.S.A. 97, 3526–3531. 10.1073/pnas.97.7.3526 10716724 PMC16273

[B24] Federle M. J. Bassler B. L. (2003). Interspecies communication in bacteria. J. Clin. Invest. 112, 1291–1299. 10.1172/JCI200320195 14597753 PMC228483

[B25] Ferluga S. Bigirimana J. Höfte M. Venturi V. (2007). A LuxR homologue of Xanthomonas oryzae pv. oryzae is required for optimal rice virulence. Mol. Plant Pathol. 8, 529–538. 10.1111/j.1364-3703.2007.00415.x 20507519

[B26] Ferluga S. Venturi V. (2009). OryR is a LuxR-family protein involved in interkingdom signaling between pathogenic Xanthomonas oryzae pv. oryzae and rice. J. Bacteriol. 191, 890–897. 10.1128/JB.01507-08 19028884 PMC2632065

[B27] Ferrer-Navarro M. Planell R. Yero D. Mongiardini E. Torrent G. Huedo P.. (2013). Abundance of the quorum-sensing factor Ax21 in four strains of *Stenotrophomonas maltophilia* correlates with mortality rate in a new zebrafish model of infection. PLoS ONE 8:e67207. 10.1371/journal.pone.0067207 23840626 PMC3693955

[B28] Fouhy Y. Scanlon K. Schouest K. Spillane C. Crossman L. Avison M. B.. (2007). Diffusible signal factor-dependent cell-cell signaling and virulence in the nosocomial pathogen *Stenotrophomonas maltophilia*. J. Bacteriol. 189, 4964–4968. 10.1128/JB.00310-07 17468254 PMC1913462

[B29] Fried L. Lassak J. Jung K. (2012). A comprehensive toolbox for the rapid construction of lacZ fusion reporters. J. Microbiol. Methods 91, 537–543. 10.1016/j.mimet.2012.09.023 23022912

[B30] Fuqua C. Greenberg E. P. (2002). Listening in on bacteria: acyl-homoserine lactone signalling. Nat. Rev. Mol. Cell Biol. 3, 685–695. 10.1038/nrm907 12209128

[B31] Fuqua C. Winans S. C. Greenberg E. P. (1996). Census and consensus in bacterial ecosystems: the LuxR-LuxI family of quorum-sensing transcriptional regulators. Annu. Rev. Microbiol. 50, 727–751. 10.1146/annurev.micro.50.1.727 8905097

[B32] Fuqua C. (2006). The QscR quorum-sensing regulon of *Pseudomonas aeruginosa*: an orphan claims its identity. J. Bacteriol. 188, 3169–3171. 10.1128/JB.188.9.3169-3171.2006 16621807 PMC1447470

[B33] Fuqua W. C. Winans S. C. (1994). A LuxR-LuxI type regulatory system activates Agrobacterium Ti plasmid conjugal transfer in the presence of a plant tumor metabolite. J. Bacteriol. 176, 2796–2806. 8188582 10.1128/jb.176.10.2796-2806.1994 PMC205432

[B34] Fuqua W. C. Winans S. C. Greenberg E. P. (1994). Quorum sensing in bacteria: the LuxR-LuxI family of cell density-responsive transcriptional regulators. J. Bacteriol. 176, 269–275. 8288518 10.1128/jb.176.2.269-275.1994 PMC205046

[B35] González J. F. Myers M. P. Venturi V. (2013). The inter-kingdom solo OryR regulator of Xanthomonas oryzae is important for motility. Mol. Plant Pathol. 14, 211–221. 10.1111/j.1364-3703.2012.00843.x 23083431 PMC6638885

[B36] Hanzelka B. L. Greenberg E. P. (1995). Evidence that the N-terminal region of the Vibrio fischeri LuxR protein constitutes an autoinducer-binding domain. J. Bacteriol. 177, 815–817. 7836318 10.1128/jb.177.3.815-817.1995 PMC176662

[B37] Hoang T. T. Karkhoff-Schweizer R. R. Kutchma A. J. Schweizer H. P. (1998). A broad-host-range Flp-FRT recombination system for site-specific excision of chromosomally-located DNA sequences: application for isolation of unmarked *Pseudomonas aeruginosa* mutants. Gene 212, 77–86. 10.1016/S0378-1119(98)00130-9 9661666

[B38] Huang T.-P. Lee Wong A. C. (2007). Extracellular fatty acids facilitate flagella-independent translocation by *Stenotrophomonas maltophilia*. Res. Microbiol. 158, 702–711. 10.1016/j.resmic.2007.09.002 18054205

[B39] Hudaiberdiev S. Choudhary K. S. Vera Alvarez R. Gelencsér Z. Ligeti B. Lamba D.. (2015). Census of solo LuxR genes in prokaryotic genomes. Front. Cell. Infect. Microbiol. 5:4. 10.3389/fcimb.2015.00004 25815274 PMC4357305

[B40] Huedo P. Yero D. Martínez-Servat S. Estibariz I. Planell R. Martínez P.. (2014). Two different rpf clusters distributed among a population of *Stenotrophomonas maltophilia* clinical strains display differential diffusible signal factor production and virulence regulation. J. Bacteriol. 196, 2431–2442. 10.1128/JB.01540-14 24769700 PMC4054175

[B43a] Jacobs M. A. Alwood A. Thaipisuttikul I. Spencer D. Haugen E. Ernst S. (2003). Comprehensive transposon mutant library of *Pseudomonas aeruginosa*. Proc. Natl. Acad. Sci. 100, 14339–14344 10.1073/pnas.2036282100 14617778 PMC283593

[B41] Kearns D. B. (2010). A field guide to bacterial swarming motility. Nat. Rev. Microbiol. 8, 634–644. 10.1038/nrmicro2405 20694026 PMC3135019

[B42] Koch B. Liljefors T. Persson T. Nielsen J. Kjelleberg S. Givskov M. (2005). The LuxR receptor: the sites of interaction with quorum-sensing signals and inhibitors. Microbiology 151, 3589–3602. 10.1099/mic.0.27954-0 16272381

[B43] Kovach M. E. Elzer P. H. Hill D. S. Robertson G. T. Farris M. A. Roop R. M.. (1995). Four new derivatives of the broad-host-range cloning vector pBBR1MCS, carrying different antibiotic-resistance cassettes. Gene 166, 175–176. 10.1016/0378-1119(95)00584-1 8529885

[B44] Lee J.-H. Lequette Y. Greenberg E. P. (2006). Activity of purified QscR, a *Pseudomonas aeruginosa* orphan quorum-sensing transcription factor. Mol. Microbiol. 59, 602–609. 10.1111/j.1365-2958.2005.04960.x 16390453

[B45] Lequette Y. Lee J.-H. Ledgham F. Lazdunski A. Greenberg E. P. (2006). A distinct QscR regulon in the *Pseudomonas aeruginosa* quorum-sensing circuit. J. Bacteriol. 188, 3365–3370. 10.1128/JB.188.9.3365-3370.2006 16621831 PMC1447466

[B46] Letunic I. Doerks T. Bork P. (2009). SMART 6: recent updates and new developments. Nucleic Acids Res. 37, D229–232. 10.1093/nar/gkn808 18978020 PMC2686533

[B47] Malki A. Caldas T. Abdallah J. Kern R. Eckey V. Kim S. J.. (2005). Peptidase activity of the *Escherichia coli* Hsp31 chaperone. J. Biol. Chem. 280, 14420–14426. 10.1074/jbc.M408296200 15550391

[B48] Malki A. Kern R. Abdallah J. Richarme G. (2003). Characterization of the *Escherichia coli* YedU protein as a molecular chaperone. Biochem. Biophys. Res. Commun. 301, 430–436. 10.1016/S0006-291X(02)03053-X 12565879

[B49] Maurhofer M. Reimmann C. Schmidli-Sacherer P. Heeb S. Haas D. Défago G. (1998). Salicylic acid biosynthetic genes expressed in pseudomonas fluorescens strain P3 improve the induction of systemic resistance in tobacco against tobacco necrosis virus. Phytopathology 88, 678–684. 10.1094/PHYTO.1998.88.7.678 18944940

[B50] Michael B. Smith J. N. Swift S. Heffron F. Ahmer B. M. (2001). SdiA of Salmonella enterica is a LuxR homolog that detects mixed microbial communities. J. Bacteriol. 183, 5733–5742. 10.1128/JB.183.19.5733-5742.2001 11544237 PMC95466

[B51] Miller J. H. (1972). Experiments in Molecular Genetics. New York, NY: Cold Spring Harbor Laboratory.

[B52] Miller M. B. Bassler B. L. (2001). Quorum sensing in bacteria. Annu. Rev. Microbiol. 55, 165–199. 10.1146/annurev.micro.55.1.165 11544353

[B53] Minogue T. D. Wehland-von Trebra M. Bernhard F. von Bodman S. B. (2002). The autoregulatory role of EsaR, a quorum-sensing regulator in Pantoea stewartii ssp. stewartii: evidence for a repressor function. Mol. Microbiol. 44, 1625–1635. 10.1046/j.1365-2958.2002.02987.x 12067349

[B54] Mitchell A. Chang H.-Y. Daugherty L. Fraser M. Hunter S. Lopez R.. (2014). The InterPro protein families database: the classification resource after 15 years. Nucleic Acids Res. 43, D213–D221. 10.1093/nar/gku1243 25428371 PMC4383996

[B55] Moskowitz S. M. Gibson R. L. Effmann E. L. (2005). Cystic fibrosis lung disease: genetic influences, microbial interactions, and radiological assessment. Pediatr. Radiol. 35, 739–757. 10.1007/s00247-005-1445-3 15868140

[B56] Mujacic M. Bader M. W. Baneyx F. (2004). *Escherichia coli* Hsp31 functions as a holding chaperone that cooperates with the DnaK-DnaJ-GrpE system in the management of protein misfolding under severe stress conditions. Mol. Microbiol. 51, 849–859. 10.1046/j.1365-2958.2003.03871.x 14731284

[B57] Mujacic M. Baneyx F. (2006). Regulation of *Escherichia coli* hchA, a stress-inducible gene encoding molecular chaperone Hsp31. Mol. Microbiol. 60, 1576–1589. 10.1111/j.1365-2958.2006.05207.x 16796689

[B58] Mujacic M. Baneyx F. (2007). Chaperone Hsp31 contributes to acid resistance in stationary-phase *Escherichia coli*. Appl. Environ. Microbiol. 73, 1014–1018. 10.1128/AEM.02429-06 17158627 PMC1800746

[B59] Oinuma K.-I. Greenberg E. P. (2011). Acyl-Homoserine lactone binding to and stability of the orphan *Pseudomonas aeruginosa* Quorum-Sensing Signal Receptor QscR. J. Bacteriol. 193, 421–428. 10.1128/JB.01041-10 21097632 PMC3019841

[B60] Overhage J. Lewenza S. Marr A. K. Hancock R. E. W. (2007). Identification of genes involved in swarming motility using a *Pseudomonas aeruginosa* PAO1 mini-Tn5-lux mutant library. J. Bacteriol. 189, 2164–2169. 10.1128/JB.01623-06 17158671 PMC1855721

[B61] Patankar A. V. González J. E. (2009). Orphan LuxR regulators of quorum sensing. FEMS Microbiol. Rev. 33, 739–756. 10.1111/j.1574-6976.2009.00163.x 19222586

[B62] Reimmann C. Ginet N. Michel L. Keel C. Michaux P. Krishnapillai V.. (2002). Genetically programmed autoinducer destruction reduces virulence gene expression and swarming motility in *Pseudomonas aeruginosa* PAO1. Microbiology 148, 923–932. 11932439 10.1099/00221287-148-4-923

[B63] Ryan R. P. Dow J. M. (2008). Diffusible signals and interspecies communication in bacteria. Microbiology 154, 1845–1858. 10.1099/mic.0.2008/017871-0 18599814

[B64] Ryan R. P. Fouhy Y. Garcia B. F. Watt S. A. Niehaus K. Yang L.. (2008). Interspecies signalling via the *Stenotrophomonas maltophilia* diffusible signal factor influences biofilm formation and polymyxin tolerance in *Pseudomonas aeruginosa*. Mol. Microbiol. 68, 75–86. 10.1111/j.1365-2958.2008.06132.x 18312265

[B65] Sambrook J. Fritsch E. F. Maniatis T. (1989). Molecular Cloning: A Laboratory Manual. Cold Spring Harbor, NY: Cold Spring Harbor Laboratory Press.

[B66] Sastry M. S. R. Korotkov K. Brodsky Y. Baneyx F. (2002). Hsp31, the *Escherichia coli* yedU gene product, is a molecular chaperone whose activity is inhibited by ATP at high temperatures. J. Biol. Chem. 277, 46026–46034. 10.1074/jbc.M205800200 12235139

[B67] Shadel G. S. Baldwin T. O. (1992). Positive autoregulation of the Vibrio fischeri luxR gene. LuxR and autoinducer activate cAMP-catabolite gene activator protein complex-independent and -dependent luxR transcription. J. Biol. Chem. 267, 7696–7702. 1373136

[B68] Shadel G. S. Young R. Baldwin T. O. (1990). Use of regulated cell lysis in a lethal genetic selection in *Escherichia coli*: identification of the autoinducer-binding region of the LuxR protein from Vibrio fischeri ATCC 7744. J. Bacteriol. 172, 3980–3987. 2141835 10.1128/jb.172.7.3980-3987.1990 PMC213383

[B69] Shaw P. D. Ping G. Daly S. L. Cha C. Cronan J. E. Rinehart K. L.. (1997). Detecting and characterizing N-acyl-homoserine lactone signal molecules by thin-layer chromatography. Proc. Natl. Acad. Sci. U.S.A. 94, 6036–6041. 10.1073/pnas.94.12.6036 9177164 PMC20996

[B70] Shiba T. Ishiguro K. Takemoto N. Koibuchi H. Sugimoto K. (1995). Purification and characterization of the *Pseudomonas aeruginosa* NfxB protein, the negative regulator of the nfxB gene. J. Bacteriol. 177, 5872–5877. 7592337 10.1128/jb.177.20.5872-5877.1995 PMC177412

[B71] Slock J. VanRiet D. Kolibachuk D. Greenberg E. P. (1990). Critical regions of the Vibrio fischeri luxR protein defined by mutational analysis. J. Bacteriol. 172, 3974–3979. 2361947 10.1128/jb.172.7.3974-3979.1990 PMC213382

[B72] Stevens A. M. Greenberg E. P. (1997). Quorum sensing in Vibrio fischeri: essential elements for activation of the luminescence genes. J. Bacteriol. 179, 557–562. 8990313 10.1128/jb.179.2.557-562.1997 PMC178731

[B73] Subedi K. P. Choi D. Kim I. Min B. Park C. (2011). Hsp31 of *Escherichia coli* K-12 is glyoxalase III. Mol. Microbiol. 81, 926–936. 10.1111/j.1365-2958.2011.07736.x 21696459

[B74] Subramoni S. Venturi V. (2009a). LuxR-family “solos”: bachelor sensors/regulators of signalling molecules. Microbiol. Read. Engl. 155, 1377–1385. 10.1099/mic.0.026849-0 19383698

[B75] Subramoni S. Venturi V. (2009b). PpoR is a conserved unpaired LuxR solo of *Pseudomonas putida* which binds N-acyl homoserine lactones. BMC Microbiol. 9:125. 10.1186/1471-2180-9-125 19534812 PMC2703642

[B76] Tamura K. Stecher G. Peterson D. Filipski A. Kumar S. (2013). MEGA6: Molecular Evolutionary Genetics Analysis version 6.0. Mol. Biol. Evol. 30, 2725–2729. 10.1093/molbev/mst197 24132122 PMC3840312

[B77] Thomas-Chollier M. Defrance M. Medina-Rivera A. Sand O. Herrmann C. Thieffry D.. (2011). RSAT 2011: regulatory sequence analysis tools. Nucleic Acids Res. 39, W86–W91. 10.1093/nar/gkr377 21715389 PMC3125777

[B78] Twomey K. B. O'Connell O. J. McCarthy Y. Dow J. M. O'Toole G. A. Plant B. J.. (2012). Bacterial cis-2-unsaturated fatty acids found in the cystic fibrosis airway modulate virulence and persistence of *Pseudomonas aeruginosa*. ISME J. 6, 939–950. 10.1038/ismej.2011.167 22134647 PMC3329111

[B79] Vannini A. Volpari C. Gargioli C. Muraglia E. Cortese R. Francesco R. D.. (2002). The crystal structure of the quorum sensing protein TraR bound to its autoinducer and target DNA. EMBO J. 21, 4393–4401. 10.1093/emboj/cdf459 12198141 PMC126196

[B80] Waters C. M. Bassler B. L. (2005). Quorum sensing: cell-to-cell communication in bacteria. Annu. Rev. Cell Dev. Biol. 21, 319–346. 10.1146/annurev.cellbio.21.012704.131001 16212498

[B81] Whitehead N. A. Barnard A. M. Slater H. Simpson N. J. Salmond G. P. (2001). Quorum-sensing in Gram-negative bacteria. FEMS Microbiol. Rev. 25, 365–404. 10.1111/j.1574-6976.2001.tb00583.x 11524130

[B82] Wopperer J. Cardona S. T. Huber B. Jacobi C. A. Valvano M. A. Eberl L. (2006). A quorum-quenching approach to investigate the conservation of quorum-sensing-regulated functions within the *Burkholderia cepacia* Complex. Appl. Environ. Microbiol. 72, 1579–1587. 10.1128/AEM.72.2.1579-1587.2006 16461713 PMC1392939

[B83] Yao Y. Martinez-Yamout M. A. Dickerson T. J. Brogan A. P. Wright P. E. Dyson H. J. (2006). Structure of the *Escherichia coli* quorum sensing protein SdiA: activation of the folding switch by acyl homoserine lactones. J. Mol. Biol. 355, 262–273. 10.1016/j.jmb.2005.10.041 16307757

[B84] Zhang R. Pappas T. Brace J. L. Miller P. C. Oulmassov T. Molyneaux J. M.. (2002). Structure of a bacterial quorum-sensing transcription factor complexed with pheromone and DNA. Nature 417, 971–974. 10.1038/nature00833 12087407

[B85] Zhu J. Chai Y. Zhong Z. Li S. Winans S. C. (2003). Agrobacterium bioassay strain for ultrasensitive detection of N-Acylhomoserine lactone-type quorum-sensing molecules: detection of autoinducers in Mesorhizobium huakuii. Appl. Environ. Microbiol. 69, 6949–6953. 10.1128/AEM.69.11.6949-6953.2003 14602662 PMC262303

[B86] Zhu J. Winans S. C. (1999). Autoinducer binding by the quorum-sensing regulator TraR increases affinity for target promoters in vitro and decreases TraR turnover rates in whole cells. Proc. Natl. Acad. Sci. U.S.A. 96, 4832–4837. 10.1073/pnas.96.9.4832 10220379 PMC21777

[B87] Zhu J. Winans S. C. (2001). The quorum-sensing transcriptional regulator TraR requires its cognate signaling ligand for protein folding, protease resistance, and dimerization. Proc. Natl. Acad. Sci. U.S.A. 98, 1507–1512. 10.1073/pnas.98.4.1507 11171981 PMC29287

