# Supplementary material for: Stenotrophomonas maltophilia responds to exogenous AHL signals through the LuxR solo SmoR (Smlt1839)
Source: Front Cell Infect Microbiol. 2015 May 15;5:41. doi: 10.3389/fcimb.2015.00041 (PMC4432800; doi:10.3389/fcimb.2015.00041)
Supplement: Supplementary file 1 [file DataSheet1.DOCX]

**Supplementary material for: Stenotrophomonas maltophilia responds to exogenous AHL signals through the LuxR solo SmoR (Smlt1839)**

**Paula Martínez^1,2#^, Pol Huedo^1,2#^, Sònia Martinez-Servat^1,2^, Raquel Planell^1^, Mario Ferrer-Navarro^1^, Xavier Daura^1,3^, Daniel Yero^1,2^* and Isidre Gibert^1,2^***

^1^Institut de Biotecnologia i de Biomedicina (IBB), Universitat Autònoma de Barcelona (UAB), Cerdanyola del Vallès (Barcelona), Spain.

^2^Departament de Genètica i de Microbiologia, Universitat Autònoma de Barcelona (UAB), Cerdanyola del Vallès (Barcelona), Spain.

^3^Catalan Institution for Research and Advanced Studies (ICREA), Barcelona, Spain.

**Supplementary material**

**

**

**Figure S1.** A) SDS-Page of protein extracts from IPTG-induced cultures of *E. coli* BL21 (DE3) harbouring either pETT22b (1) or pET22b-SmoR (2). M: Ladder; *: protein band corresponding to overexpressed SmoR. B) MALDI-MS chromatogram showing peaks corresponding to SmoR peptides generated after trypsin digestion. Underlined sequences correspond to identified peptides.
